# Procalcitonin Monitoring and Antibiotic Duration in Presumed Lower Respiratory Tract Infections: A Propensity Score–Matched Cohort Across the Veterans Health Administration

**DOI:** 10.1093/ofid/ofad520

**Published:** 2023-10-25

**Authors:** Jamie E Heren, Brian C Lund, Bruce Alexander, Daniel J Livorsi

**Affiliations:** Center for Access & Delivery Research & Evaluation (CADRE), Iowa City Veterans Affairs Health Care System, Iowa City, Iowa, USA; Center for Access & Delivery Research & Evaluation (CADRE), Iowa City Veterans Affairs Health Care System, Iowa City, Iowa, USA; Center for Access & Delivery Research & Evaluation (CADRE), Iowa City Veterans Affairs Health Care System, Iowa City, Iowa, USA; Center for Access & Delivery Research & Evaluation (CADRE), Iowa City Veterans Affairs Health Care System, Iowa City, Iowa, USA; Division of Infectious Diseases, University of Iowa Carver College of Medicine, Iowa City, Iowa, USA

**Keywords:** antibiotic use, hospitals, procalcitonin, respiratory infections

## Abstract

**Background:**

Randomized controlled trials have shown that procalcitonin-guided algorithms can reduce antibiotic duration for lower respiratory tract infections (LRTIs). The goal of this study was to compare antibiotic duration for LRTIs with and without procalcitonin testing in real-life practice.

**Methods:**

This retrospective cohort study included all acute care hospital admissions for presumed LRTIs between 1/2018 and 12/2021 at 81 Veterans Affairs facilities with on-site procalcitonin testing. The exposure was procalcitonin testing; the primary outcome was antibiotic duration. We used 1:1 nearest-neighbor propensity score matching to estimate the difference in outcome between procalcitonin-tested and nontested patients.

**Results:**

A total of 35 610 patients with LRTIs were included (6015 [16.9%] with procalcitonin testing; 29 595 [83.1%] without testing). In tested patients, the median number of procalcitonin levels checked (interquartile range) was 2 (1–3). The mean antibiotic duration was 10.0 days in the procalcitonin group compared with 8.3 days in nontested patients (unadjusted difference, 1.7 days; *P* < .0001). After propensity score matching with 3903 pairs, antibiotic duration remained greater in the procalcitonin group (9.6 days vs 9.2 days; *P* < .0001). In a subgroup analysis of 2241 tested patients with a procalcitonin value at the standard threshold for antibiotic discontinuation, antibiotic duration was shorter in tested vs nontested patients, with a mean difference of 0.1 days (*P* < .01).

**Conclusions:**

In this retrospective propensity-matched cohort of patients with presumed LRTIs across a geographically diverse group of hospitals, patients who underwent procalcitonin testing did not have a meaningful reduction in antibiotic duration compared with those who were not tested. Poor implementation of procalcitonin testing may have undermined its effectiveness.

## Teaser

Across an integrated healthcare network, procalcitonin monitoring was not associated with a meaningful reduction in antibiotic duration among patients hospitalized with presumed lower respiratory tract infections. Poor implementation of the procalcitonin test may explain its apparent lack of benefit.

One of every 2 hospitalized patients will receive an antibiotic [1]. Decision-making about how and when to prescribe antibiotics to hospitalized patients can be challenging [2]. Given the public health crisis of antibiotic resistance, there is a need to improve antibiotic prescribing, and tools that can aid in the optimization of antibiotic use are needed.

Procalcitonin, an inflammatory biomarker that is upregulated in response to bacterial infections, has the potential to guide the prescription of antibiotics. The procalcitonin test is cleared by the Food and Drug Administration (FDA) for use in several clinical scenarios, including as a guide to decision-making about initiating and discontinuing antibiotics for patients with lower respiratory tract infections (LRTIs) [3]. Several randomized controlled trials and a large meta-analysis have demonstrated lower rates of antibiotic exposure with procalcitonin-guided treatment of LRTIs [4–7]. However, real-world studies on whether procalcitonin testing helps decrease antibiotic duration have reached conflicting results [8–11].

Understanding the effectiveness of procalcitonin is important because use of this test is increasing in some clinical settings. For example, between 2015 and 2020, the number of acute care hospitals with on-site procalcitonin testing increased from 20% to 65% across the Veterans Health Administration, the largest integrated health care system in the United States [12, 13]. The purpose of this study was to estimate the difference in antibiotic duration between procalcitonin-tested and nontested patients who were hospitalized with presumed LRTIs. By focusing on antibiotic duration, this study will be assessing the use of procalcitonin to specifically inform decisions about antibiotic discontinuation.

## METHODS

### Patient Consent

This study was approved by the University of Iowa Institutional Review Board and the Iowa City Veterans Administration Research and Development Committee. The study followed the Strengthening the Reporting of Observational Studies in Epidemiology (STROBE) reporting guidelines (Supplementary Table 1).

### Design and Data Sources

We performed a retrospective cohort study with propensity score matching to determine whether procalcitonin testing among inpatients with presumed LRTIs was associated with shorter durations of antibiotic therapy under real-life circumstances, compared with patients who did not receive this testing.

We accessed national administration data from the Veterans Administration (VA) Corporate Data Warehouse via the VA Informatics and Computing Infrastructure. Acute inpatient hospitalizations were identified using inpatient encounter data. Inpatient and outpatient pharmacy dispensing data were used to ascertain antibacterial medication exposure during the course of hospitalization and the antibiotic regimen dispensed at discharge. Laboratory data were used to identify procalcitonin testing and assess analysis covariates such as white blood cell counts and serum creatinine. Other covariate information was obtained from vital sign data (eg, blood pressure and pulse), and medical comorbidities were assessed using International Classification of Diseases, 10th revision (ICD-10), coded inpatient and outpatient encounter data. Data on the availability of on-site procalcitonin testing, antibiotic stewardship resources, and processes were obtained from a mandatory survey of all VA hospitals conducted between 10/20/2020 and 11/10/2020.

### Patients

The target population was all hospital admissions for presumed LRTIs at VA acute care facilities that were ordering on-site procalcitonin testing with some degree of frequency between January 2018 and December 2021. To define eligible sites, we identified hospitals that reported use of procalcitonin testing on the above-mentioned survey and determined the rate of testing per acute care admissions per month among these facilities, with a benchmark set at the 25th percentile. Hospitals with at least 12 months of observed testing rates above this benchmark, regardless of reported use on the above-mentioned survey, were selected for the analysis. We chose to limit our analysis to these hospitals because we speculated that the benefits of procalcitonin testing would most likely be realized in settings with more experience interpreting the test.

Hospital encounters for presumed LRTIs were identified by either (1) ICD-10 codes for pneumonia or acute exacerbations of chronic obstructive pulmonary disease (COPD) during the hospital stay or at hospital discharge, as previously defined [14]; or (2) receipt of an antibacterial regimen specific to community-acquired pneumonia (eg, a macrolide plus an antipneumococcal beta-lactam) during the first 48 hours of the treatment course (Supplementary Table 2). Cases of pneumonia were classified as community-acquired vs hospital-acquired based on the timing of antibacterial initiation (≤48 hours from admission vs >48 hours after admission, respectively).

To ensure that the treating provider had a strong suspicion for an LRTI, hospital admissions were excluded if the patient received inpatient antibacterials for ≤48 hours. Additional exclusion criteria were as follows: the patient received an antibacterial agent that is not normally used to treat pneumonia; there was evidence of a concurrent nonrespiratory tract infection based on ICD-10 codes; the patient was transferred to the VA from another hospital; the patient was admitted for ≥30 days; a procalcitonin value was checked during the initial 48 hours of antibacterial therapy without any repeat measurements after 48 hours of antibacterials; or the episode of pneumonia had evidence of complicating factors, defined as (a) having a diagnosis of pneumonia within the prior 30 days; (b) ICD-10 codes from the admission indicated the presence of a lung abscess, an empyema, or lung necrosis; or (c) a pleural drainage procedure was performed during the hospitalization or the prior 30 days (Supplementary Table 2).

### Outcomes

The primary outcome was length of antibiotic therapy, defined as the number of unique calendar days a patient received an antibacterial agent, regardless of the number of different drugs administered. Length of therapy included the sum of both inpatient and postdischarge antibiotic therapy. Postdischarge therapy was defined as previously described [15]. Secondary outcomes were chosen to describe additional markers of antibiotic duration as well as potential antibiotic-associated complications. Days of therapy was defined as the aggregate sum of days for which any amount of a specific antibiotic agent was administered. Patients were considered to have antibiotic-associated diarrhea if they had a stool sample collected for *Clostridioides difficile* testing on or after hospital day 4 and within 30 days of hospital discharge; patients were considered to have *C*. *difficile* infection (CDI) if they received an antibiotic for CDI (oral vancomycin, metronidazole, or fidaxomicin) within 72 hours of a positive *C*. *difficile* test result. Hospital readmission and/or death was measured as a composite outcome within 30 days of the patient's discharge.

### Statistical Analysis

Given the strong potential for selection bias, we used 1:1 nearest-neighbor propensity score matching to estimate the difference in the primary outcome between procalcitonin-tested and nontested patients. Procalcitonin-tested patients were matched to nontested patients on the hospital day that the first eligible procalcitonin test was performed, assuming the patient had already received at least 48 hours of inpatient antibiotics. To ensure that matched patients would be exposed to similar clinical care (eg, stewardship processes), patients were matched on hospital site and admission date (within 180 days) in addition to being matched on their propensity score. Propensity scores for the likelihood of receiving procalcitonin testing were computed for each patient using a logistic regression model with the following covariates: age, sex, race, infection type (ie, COPD exacerbation, hospital-acquired pneumonia, and community-acquired pneumonia), certain comorbidities, immunosuppression, risk factors for antibiotic resistance, methicillin-resistant *Staphylococcus aureus* nasal colonization, coronavirus disease 2019 (COVID-19) status, urine *Legionella* antigen results, a modified Acute Physiology and Chronic Health Evaluation (APACHE) score calculated at the time of admission, and several variables measured at the time point of matching: hospital unit type, vital signs, white blood cell count, serum creatinine, and antibiotic class. Laboratory data and vital signs on the day of matching were missing for ∼15%–25% of patients in the final postmatch cohort. Values from prior days of hospitalization were used based on a last-observation-carried-forward approach. A more detailed description of covariates used for the propensity score can be found in Supplementary Table 3. Matched controls were required to score within a caliper of 0.2 (ie, 0.2 times the standard deviation of the logit of the propensity scores), relative to cases. Standardized differences were calculated before and after matching to assess balance in baseline characteristics between groups. Discrete outcome measures were contrasted between procalcitonin cases and matched controls using the Wilcoxon signed rank test and McNemar's test for dichotomous variables.

Within the propensity-matched cohort, a subgroup analysis was performed among tested patients (and their matched controls) if the tested patient had a procalcitonin value that met the standard threshold for antibiotic discontinuation: ≤0.25 ng/mL or >80% decrease in procalcitonin over the course of antibiotic therapy [8]. In addition, to see whether more frequent procalcitonin use was associated with greater effectiveness of the test, a stratified analysis was performed among tested patients (and their matched controls) within the 3 tertiles of hospital-level procalcitonin use. At the hospital level, procalcitonin use was defined by the mean number of tests ordered per admission per month.

## RESULTS

Eighty-one VA medical centers had procalcitonin testing available. These sites spanned all 4 US Census Regions, including 31 in the South, 23 in the Midwest, 19 in the West, and 8 in the Northeast. The median number of acute care beds per hospital (interquartile range [IQR]) was 79 (41–111). Hospitals with procalcitonin testing generally had robust stewardship resources and processes; these hospitals differed in some ways from the 44 hospitals without on-site procalcitonin testing (Supplementary Table 4). On average, VA medical centers with available procalcitonin testing ordered the test 60.9 times per month for hospitalized patients.

**Figure 1.**
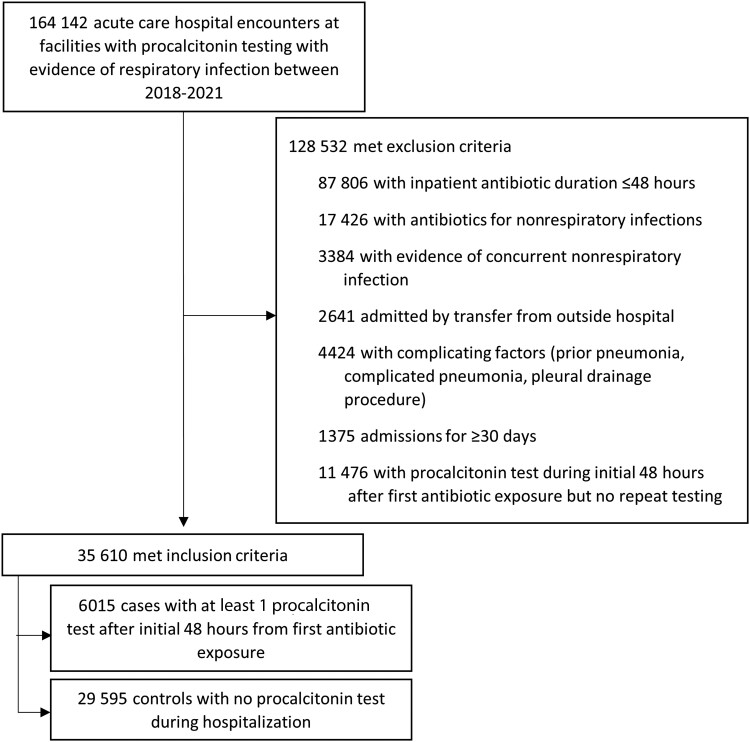
Flow diagram for the study cohort.

A total of 164 142 patients were hospitalized for a presumed LRTI at the 81 sites with procalcitonin testing. Of the 35 610 encounters that met inclusion criteria, 6015 (16.9%) had ≥1 procalcitonin test at least 48 hours into their antibiotic course, and 29 595 (83.1%) did not have any procalcitonin testing during their hospital admission (Figure 1).

For patients included in the procalcitonin testing group (n = 6015), the median number of procalcitonin tests checked during the entire antibiotic course (IQR) was 2 (1–3), and 58.2% had a procalcitonin value checked during the initial 48 hours of antibiotics. Among procalcitonin values checked after the first 48 hours of antibiotics, there were 3435 (57.1%) patients who had a procalcitonin value that met the standard threshold for antibiotic discontinuation (ie, procalcitonin ≤0.25 ng/mL or >80% decrease from their maximum procalcitonin value): 1057 (30.8%) of these patients had antibiotics stopped within 24 hours of meeting this threshold, while 1737 (50.6%) remained on antibiotics for >24 hours after the threshold was reached. For the latter category, the mean duration of excess antibiotics after meeting the discontinuation threshold (SD) was 3.2 (2.7) days. The remaining 641 (18.7%) patients had antibiotics stopped >24 hours before the procalcitonin threshold for antibiotic discontinuation was met.

Baseline characteristics for patients with procalcitonin testing or no procalcitonin testing are given in Table 1. Before matching, standardized differences >0.10 between procalcitonin-tested and nontested patients were observed for several clinical characteristics, including intensive care unit status (35.7% vs 14.3%), diagnosis of community-acquired pneumonia (80.7% vs 64.7%) or hospital-acquired pneumonia (8.5% vs 4.1%), positive test for COVID-19 (10.3% vs 1.1%), and treatment with a cephalosporin (43.8% vs 35.4%), beta-lactam/beta-lactamase inhibitor combination (33.2% vs 23.2%), or glycopeptide (24.3% vs 10.4%) antibiotic.

**Table 1.** Baseline Characteristics of Procalcitonin-Tested and Nontested Patients Hospitalized With a Presumed Lower Respiratory Tract Infection at 81 VA Medical Centers, 2018–2021

	Overall Series			Propensity Score–Matched Pairs		
	Procalcitonin-Tested (n = 6015)	Not Tested (n = 29 595)	Standard Difference	Procalcitonin-Tested (n = 3903)	Not Tested (n = 3903)	Standard Difference
Age, mean (SD), y	72.5 (11.3)	72.6 (10.5)	0.009	72.7 (11.1)	72.7 (11.1)	0
Female, No. (%)	183 (3.0)	1247 (4.2)	0.063	134 (3.4)	147 (3.8)	0.018
Race, No. (%)						
White	4330 (72.0)	22 704 (76.7)	0.108	2906 (74.5)	2988 (76.6)	0.049
Black	1450 (24.1)	6041 (20.4)	0.089	869 (22.3)	795 (20.4)	0.046
Other	235 (3.9)	850 (2.9)	0.057	128 (3.3)	120 (3.1)	0.012
ICU status, No. (%)	2147 (35.7)	4224 (14.3)	0.511	985 (25.2)	902 (23.1)	0.050
Infection type, No. (%)						
COPD exacerbation	649 (10.8)	9234 (31.2)	0.518	556 (14.2)	583 (14.9)	0.020
Community-acquired pneumonia	4852 (80.7)	19 136 (64.7)	0.365	3098 (79.4)	3069 (78.6)	0.018
Hospital-acquired pneumonia	514 (8.5)	1225 (4.1)	0.182	249 (6.4)	251 (6.4)	0.002
Clinical parameters, mean (SD)						
Systolic blood pressure, mmHg	141 (22.1)	143 (20.7)	0.098	142 (21.5)	142 (21.1)	0.009
Heart rate, beats per minute	92 (18.2)	90 (16.5)	0.109	92 (17.6)	90 (17.1)	0.063
Respiration rate, breaths per minute	21 (4.4)	20 (3.1)	0.263	21 (3.6)	20 (3.5)	0.056
Temperature, °F	98.6 (1.1)	98.4 (0.8)	0.208	98.5 (1.0)	98.5 (0.9)	0
Laboratory findings						
White blood cells, mean (SD), ×10^3^/mm^3^	10.5 (6.4)	9.5 (5.3)	0.170	10.2 (5.7)	9.8 (6.1)	0.068
Serum creatinine, mean (SD), mg/dL	1.6 (1.5)	1.3 (1.1)	0.228	1.4 (1.3)	1.4 (1.4)	0
MRSA positive, No. (%)	480 (8.0)	1878 (6.3)	0.063	290 (7.4)	266 (6.8)	0.024
*Legionella* positive, No. (%)	16 (0.3)	52 (0.2)	0.019	8 (0.2)	8 (0.2)	0
COVID positive, No. (%)	619 (10.3)	327 (1.1)	0.404	135 (3.5)	116 (3.0)	0.028
Comorbidities, No. (%)						
Alcohol use disorder	885 (14.7)	5234 (17.7)	0.081	601 (15.4)	607 (15.6)	0.004
Congestive heart failure	2629 (43.7)	12 979 (43.9)	0.003	1705 (43.7)	1683 (43.1)	0.011
COPD	3756 (62.4)	22 486 (76.0)	0.296	2598 (66.6)	2556 (65.5)	0.023
Malignancy	1706 (28.4)	8078 (27.3)	0.024	1142 (29.3)	1180 (30.2)	0.021
Dementia	918 (15.3)	3926 (13.3)	0.057	590 (15.1)	580 (14.9)	0.007
Diabetes	2792 (46.4)	13 133 (44.4)	0.041	1751 (44.9)	1778 (45.6)	0.014
Immunosuppressed	766 (12.7)	2924 (9.9)	0.090	441 (11.3)	460 (11.8)	0.015
Rheumatic disease	228 (3.8)	1012 (3.4)	0.020	140 (3.6)	142 (3.6)	0.003
Risk factors for antibiotic resistance, No. (%)						
Wound care	3 (<0.1)	5 (<0.1)	0.018	1 (<0.1)	2 (0.1)	0.013
Dialysis	181 (3.0)	321 (1.1)	0.136	54 (1.4)	78 (2.0)	0.048
Prior admission	1470 (24.4)	8124 (27.5)	0.069	1010 (25.9)	1037 (26.6)	0.016
Admission from long-term care facility	116 (1.9)	387 (1.3)	0.049	59 (1.5)	80 (2.0)	0.041
Antibiotics, No. (%)						
Cephalosporin	2635 (43.8)	10 465 (35.4)	0.173	1674 (42.9)	1726 (44.2)	0.027
Beta-lactam/beta-lactamase inhibitor	1997 (33.2)	6858 (23.2)	0.224	1259 (32.3)	1038 (26.6)	0.124
Carbapenem	223 (3.7)	427 (1.4)	0.143	96 (2.5)	121 (3.1)	0.039
Fluoroquinolone	493 (8.2)	3470 (11.7)	0.118	358 (9.2)	326 (8.4)	0.029
Glycopeptide	1460 (24.3)	3085 (10.4)	0.372	700 (17.9)	706 (18.1)	0.004
Macrolide	2040 (33.9)	11 578 (39.1)	0.108	1411 (36.2)	1289 (33.0)	0.066
Tetracycline	738 (12.3)	4093 (13.8)	0.046	480 (12.3)	538 (13.8)	0.044
Other	211 (3.5)	1021 (3.4)	0.003	139 (3.6)	160 (4.1)	0.028
Index for disease severity						
Modified APACHE II score, mean (SD)	42.4 (20.2)	39.7 (16.9)	0.145	41.3 (19.3)	41.4 (18.1)	0.005

Data are presented as mean (SD) or No. (%).

Abbreviations: APACHE, Acute Physiology and Chronic Health Evaluation; ICU, intensive care unit; COPD, chronic obstructive pulmonary disease; MRSA, methicillin-resistant *Staphylococcus aureus*.

Before propensity score matching, the mean length of antibiotic therapy was 10.0 days in the patients with procalcitonin testing compared with 8.3 days in patients without testing (unadjusted difference, 1.7 days; *P* < .01). Compared with patients without procalcitonin testing, patients in the procalcitonin-tested group had a longer length of therapy in the inpatient setting (8.2 days vs 5.5 days) but a shorter length of therapy on discharge (1.8 days vs 2.8 days). Hospital readmission and/or death occurred in 29.4% of procalcitonin-tested patients and 17.8% of untested patients (unadjusted odds ratio [OR], 1.93; 95% CI, 1.81–2.06).

Using propensity scores, 3903 encounters in the procalcitonin-tested group were matched to 3903 patients in the nontested group (Table 1). The remaining 2112 patients from the procalcitonin group failed to satisfy matching criteria with any control patient and were not included in the propensity-matched analysis. In the propensity scored–matched cohort, the length of therapy was higher in the procalcitonin group compared with the nontested group, with a median (IQR) of 8 (6–11) days compared with 7 (5–10) days and an unadjusted mean difference of 0.4 days (*P* < .01) (Table 2). Durations >5 days were prescribed in 79.0% of procalcitonin patients and 72.6% of nontested patients (chi-square = 46.1; *P* < .001), and durations >7 days were prescribed to 55.3% and 45.5% of tested and nontested patients, respectively (chi-square = 66.1; *P* < .001). The procalcitonin-tested group also had more days of antibiotic therapy (unadjusted mean difference, 1.1 days; *P* < .01) and an increased odds of antibiotic-associated diarrhea (OR, 1.48; 95% CI, 1.26–1.74) but similar rates of *C. difficile* infection (OR, 1.23; 95% CI, 0.76–2.00). In addition, the procalcitonin-tested group had increased odds of hospital readmission and/or death (OR, 1.34; 95% CI, 1.21–1.49).

**Table 2.** Clinical Outcomes for Propensity-Matched Cohort of Patients Hospitalized With a Presumed Lower Respiratory Tract Infection at 81 VA Medical Centers, 2018–2021

	Procalcitonin-Tested (n = 3903)	Not Tested (n = 3903)	Difference	*P* Value	OR (95% CI)
Primary outcome					
Length of therapy,^a^ mean (SD), d	9.6 (7.2)	9.2 (8.9)	0.4	<.0001	
Secondary outcomes					
Days of therapy, mean (SD)	14.4 (9.3)	13.3 (10.9)	1.1	<.0001	
Length of acute care stay, mean (SD), d	11.1 (6.3)	8.3 (5.0)	2.8	<.0001	
Antibiotic-associated diarrhea, No. (%)	392 (10.0)	275 (7.0)			1.48 (1.26–1.74)
* C. difficile* infection, No. (%)	38 (1.0)	31 (0.8)			1.23 (0.76–2.00)
Hospital readmission and/or death, No. (%)	1043 (26.7)	840 (21.5)			1.34 (1.21–1.49)

Continuous data are expressed as mean (standard deviation) with calculated difference. Frequency data are expressed as number (%) with odds ratios.

Abbreviations: IQR, interquartile range; OR, odds ratio; VA, Veterans Administration.

^a^The median (IQR) for the case and control groups is 8 days (6–11) and 7 days (5–10), respectively.

Within the propensity-matched cohort, there were 2241 patients who either had a final procalcitonin value ≤0.25 or a decrease in procalcitonin >80%. Compared with their 2241 matched controls, the length of antibiotic therapy was significantly shorter in the procalcitonin-tested group, with a mean difference of 0.1 days (9.3 vs 9.4 days; *P* < .01).

In the analysis stratified based on hospital-level procalcitonin use, there were 1792 patients who were managed at a high-use hospital, 1422 at a medium-use hospital, and 689 at a low-use hospital. Compared with their matched controls, length of antibiotic therapy was significantly longer in the procalcitonin-tested group in the medium- (9.6 vs 9.3 days; *P* < .01) and high-use hospitals (9.5 vs 8.8 days; *P* < .01). In the low-use hospitals, there was no significant difference in length of therapy between tested patients and their controls (9.7 vs 10.2 days; *P* = .23).

## DISCUSSION

In this retrospective propensity-matched cohort of patients with presumed LRTIs across 81 hospitals, we failed to demonstrate a clinically meaningful reduction in antibiotic duration for patients who underwent procalcitonin monitoring compared with those who did not. This finding, which reflects procalcitonin use under real-life circumstances, calls into question the effectiveness of procalcitonin testing to decrease antibiotic duration for LRTIs. This lack of effectiveness may be the result of testing practices that differed from how procalcitonin has been studied. In these hospitals, the test was not ordered routinely on eligible patients, procalcitonin levels were not serially monitored, and standard procalcitonin thresholds for stopping antibiotics were frequently not followed.

Our findings contrast with the majority of the published clinical trials on procalcitonin use in acute respiratory infections. For example, a meta-analysis of 24 largely European randomized controlled trials found that measurement of procalcitonin in acute respiratory infections reduced antibiotic exposure by 2.4 days (8.1 vs 5.7 days) [4]. However, in a more recent multicenter randomized controlled trial of patients presenting to emergency departments at US hospitals for a presumed LRTI, there was no significant difference in the antibiotic length of therapy or antibiotic-related adverse events with a procalcitonin-guided protocol [16]. Some real-world studies have shown that procalcitonin use helps decrease antibiotic duration for LRTIs [8, 9]. Other real-world studies on procalcitonin testing have failed to show a reduction in antibiotic use [10, 11].

One reason our findings may differ from other literature on procalcitonin testing is that we limited our analysis to patients who had already received at least 48 hours of antibiotics. As a result, the potential impact of stopping antibiotics before 48 hours or never starting them at all could not be measured. This may have limited the test's overall effect on antibiotic exposure for presumed LRTIs.

The failure to see a meaningful difference in antibiotic exposure with procalcitonin monitoring may also reflect poor implementation of the test. Our data indicate that procalcitonin was not typically being ordered in a serial fashion (eg, every 1–2 days), which is in contrast to its use in clinical trials [5–8]. Furthermore, in the patients who did have documentation of low or declining procalcitonin values, only 1 out of every 2 patients had antibiotics discontinued. Even patients at hospitals that more frequently used procalcitonin testing did not see a meaningful reduction in antibiotic duration. In prior studies, high compliance to a validated procalcitonin algorithm was necessary to realize a benefit from the test [8, 17]. But in line with our findings, 2 observational studies across large US health care systems also found poor implementation of the test, including infrequent serial monitoring of procalcitonin levels and a failure to stop antibiotics even when procalcitonin thresholds for discontinuation were met [10, 18].

Despite a large body of literature attesting to the efficacy of procalcitonin testing in patients with LRTIs, it is surprising that procalcitonin levels were not more frequently checked among the patients we identified. Overall, ∼1 in 6 patients with a presumed LRTI had a procalcitonin level measured >48 hours after antibiotic initiation. It is unclear if such low usage reflects clinicians’ lack of awareness of the test or a lack of enthusiasm for the test's utility. Our findings indicate that clinicians are using the procalcitonin test more often in patients who have a more severe illness, as suggested by the longer length of stay and higher hospital readmission/mortality rate in tested patients. In these situations, clinicians may have been looking for additional data points (eg, a procalcitonin value) to guide their decision-making.

A notable feature of our cohort was the robustness of reported antibiotic stewardship resources and processes at the included hospitals. However, it is unclear whether these stewardship processes incorporated procalcitonin monitoring. Even in the presence of this stewardship expertise, there may still be room for further reduction in antibiotic duration for presumed LRTIs. An antibiotic duration of 5 days should be adequate for most cases of community-acquired pneumonia and acute exacerbations of COPD [19, 20]. But in our cohort, the median duration of therapy for procalcitonin-tested and nontested patients was 8 and 7 days, respectively. This was comparable to the median length of therapy (7.7 days) across a non-VA cohort of US patients aged ≥65 years hospitalized with pneumonia in 2020 [21].

To our knowledge, this is only the second assessment of the association between procalcitonin monitoring and antibiotic duration in a geographically diverse group of US hospitals. While the prior study focused on intensive care unit patients with sepsis, the present study looked specifically at patients with LRTIs [10]. The present study has the added strength of minimizing the effect of selection bias through its use of propensity score matching.

This study also has some limitations that need to be acknowledged. First, there could still be unmeasured confounding between procalcitonin-tested and nontested patients even though our propensity score matching was comprehensive. For example, antibiotic agents were grouped into broad categories to calculate propensity scores, and this may have overlooked differences in treatment regimens. There may also have been interprovider variability in both procalcitonin testing and antibiotic use, which we were unable to account for in our analysis. Residual confounding could explain why patients who had procalcitonin monitoring also had more adverse outcomes than patients who were not tested. Second, we were unable to differentiate antibiotic use intended to treat LRTIs from antibiotic use for nonrespiratory infections. To minimize this concern, we eliminated patients with diagnostic codes for nonrespiratory infections and patients who received antibiotics not intended for respiratory infections, but nevertheless, some other infections may still have been missed. Third, we relied on diagnostic codes and, in some cases, antibiotic selection to characterize clinical suspicion for LRTIs without a more in-depth validation of this diagnosis. While patients without confirmed LRTIs may have been inadvertently included in our analysis, this misclassification was likely evenly distributed between cases and controls. Fourth, we only captured certain outcomes identified within VA medical centers, so the results for hospital readmission, antibiotic-associated diarrhea, and *C. difficile* infections may be an underestimate if patients sought follow-up care at non-VA facilities. This bias, however, should have been nondifferential. Fifth, our cohort included far more cases of community-acquired pneumonia than were included in published randomized controlled trials on procalcitonin use for LRTIs [4, 16]. It is unclear how this unique feature of our study would have affected the findings. Finally, this study may not be generalizable to all hospitalized patients with pneumonia because our propensity-matched cohort was only a subset of the total number of cases who had this condition. While this study may also not be generalizable to non-VA settings, the large number of included hospitals and their geographic diversity should improve the external validity of our findings.

In conclusion, the results of this retrospective cohort study fail to support a clinically meaningful reduction in antibiotic duration for hospitalized patients with presumed LRTIs monitored with procalcitonin on or after day 3 of therapy. Potential benefits of procalcitonin monitoring across these 81 hospitals may have been negated by poor implementation of the test.

## Supplementary Material

ofad520_Supplementary_Data

## Supplementary Data

Supplementary materials are available at *Open Forum Infectious Diseases* online. Consisting of data provided by the authors to benefit the reader, the posted materials are not copyedited and are the sole responsibility of the authors, so questions or comments should be addressed to the corresponding author.
