# Supplementary material for: Procalcitonin Monitoring and Antibiotic Duration in Presumed Lower Respiratory Tract Infections: A Propensity Score–Matched Cohort Across the Veterans Health Administration
Source: Open Forum Infect Dis. 2023 Oct 25;10(11):ofad520. doi: 10.1093/ofid/ofad520 (PMC10629903; doi:10.1093/ofid/ofad520)
Supplement: ofad520_Supplementary_Data [file ofad520_supplementary_data.docx]

**Supplemental Table 1. STROBE Statement—Checklist of items that should be included in reports of observational studies**

|  | | Item No | Recommendation | Page No | |
| --- | --- | --- | --- | --- | --- |
| **Title and abstract** | | 1 | (*a*) Indicate the study’s design with a commonly used term in the title or the abstract | 1 | |
|  |  |  | (*b*) Provide in the abstract an informative and balanced summary of what was done and what was found | 3-4 | |
| Introduction | | | | | |
| Background/rationale | | 2 | Explain the scientific background and rationale for the investigation being reported | 5 | |
| Objectives | | 3 | State specific objectives, including any prespecified hypotheses | 5 | |
| Methods | | | | | |
| Study design | | 4 | Present key elements of study design early in the paper | 5-7 | |
| Setting | | 5 | Describe the setting, locations, and relevant dates, including periods of recruitment, exposure, follow-up, and data collection | 6 | |
| Participants | | 6 | (*a*) Give the eligibility criteria, and the sources and methods of case ascertainment and control selection. Give the rationale for the choice of cases and controls | 6-7 | |
|  |  |  | (*b*) For matched studies, give matching criteria and the number of controls per case | 7-8 | |
| Variables | | 7 | Clearly define all outcomes, exposures, predictors, potential confounders, and effect modifiers. Give diagnostic criteria, if applicable | 7 | |
| Data sources/ measurement | | 8* | For each variable of interest, give sources of data and details of methods of assessment (measurement). Describe comparability of assessment methods if there is more than one group | 6 | |
| Bias | | 9 | Describe any efforts to address potential sources of bias | 7-8 | |
| Study size | | 10 | Explain how the study size was arrived at | 6 | |
| Quantitative variables | | 11 | Explain how quantitative variables were handled in the analyses. If applicable, describe which groupings were chosen and why | 7-8 | |
| Statistical methods | | 12 | (*a*) Describe all statistical methods, including those used to control for confounding | 7-8 | |
|  |  |  | (*b*) Describe any methods used to examine subgroups and interactions | 8 | |
|  |  |  | (*c*) Explain how missing data were addressed | 8 | |
|  |  |  | (*d*) If applicable, explain how loss to follow-up was addressed | 7-8 | |
|  |  |  | (*e*) Describe any sensitivity analyses | N/A | |
| Results | | | | | |
| Participants | | 13* | (a) Report numbers of individuals at each stage of study—eg numbers potentially eligible, examined for eligibility, confirmed eligible, included in the study, completing follow-up, and analysed | Fig 1 | |
|  |  |  | (b) Give reasons for non-participation at each stage | Fig 1 | |
|  |  |  | (c) Consider use of a flow diagram | Fig 1 | |
| Descriptive data | | 14* | (a) Give characteristics of study participants (eg demographic, clinical, social) and information on exposures and potential confounders | Table 1 | |
|  |  |  | (b) Indicate number of participants with missing data for each variable of interest | 8, Supplemental Table 3 | |
|  |  |  | (c ) Summarise follow-up time (e.g., average and total amount) | Table 2 | |
| Outcome data | | 15* | Report numbers of outcome events or summary measures over time | 9-10 | |
| Main results | | 16 | (*a*) Give unadjusted estimates and, if applicable, confounder-adjusted estimates and their precision (eg, 95% confidence interval). Make clear which confounders were adjusted for and why they were included | 9-10 |  |
|  |  |  | (*b*) Report category boundaries when continuous variables were categorized | 9-10 |  |
|  |  |  | (*c*) If relevant, consider translating estimates of relative risk into absolute risk for a meaningful time period | N/A |  |
| Other analyses | 17 | Report other analyses done—eg analyses of subgroups and interactions, and sensitivity analyses | | 10 |  |
| Discussion | | | | |  |
| Key results | 18 | Summarise key results with reference to study objectives | | 10-11 |  |
| Limitations | 19 | Discuss limitations of the study, taking into account sources of potential bias or imprecision. Discuss both direction and magnitude of any potential bias | | 12-13 |  |
| Interpretation | 20 | Give a cautious overall interpretation of results considering objectives, limitations, multiplicity of analyses, results from similar studies, and other relevant evidence | | 13 |  |
| Generalisability | 21 | Discuss the generalisability (external validity) of the study results | | 13 |  |
| Other information | | | | |  |
| Funding | 22 | Give the source of funding and the role of the funders for the present study and, if applicable, for the original study on which the present article is based | | 13 |  |

*Give information separately for cases and controls.

**Note:** An Explanation and Elaboration article discusses each checklist item and gives methodological background and published examples of transparent reporting. The STROBE checklist is best used in conjunction with this article (freely available on the Web sites of PLoS Medicine at http://www.plosmedicine.org/, Annals of Internal Medicine at http://www.annals.org/, and Epidemiology at http://www.epidem.com/). Information on the STROBE Initiative is available at http://www.strobe-statement.org.

**Supplemental Table 2. Inclusion and exclusion criteria for the study cohort**

| **Inclusion criteria** | **Description** |
| --- | --- |
| 1 | Acute-care hospitalization at a VA medical center during 2018-2021 |
| 2 | On-site procalcitonin was available at the hospital and was being ordered frequently^1^ |
| 3 | 1. diagnosis of pneumonia or an acute exacerbation of COPD during the hospital stay or at discharge,^2^ or b) receipt of greater than 48 hours of an antibacterial regimen specific for community-acquired pneumonia^3^ |
| **Exclusion criteria** | **Description** |
| 1 | Duration of inpatient antibiotics was < 48 hours |
| 2 | Administration of an antibacterial not routinely used to treat bacterial pneumonia^4^ |
| 3 | Evidence of a concurrent non-respiratory tract infection based on ICD-10 codes^5^ |
| 4 | Transferred to the VA from another hospital |
| 5 | Acute-care stay lasted ≥ 30 days |
| 6 | Procalcitonin value was checked during the first 48 hours of antibacterial administration but not rechecked after the 48 hours of antibacterial. |
| 7 | Pneumonia was complicated, as suggested by a) a diagnosis of pneumonia in the prior 30 days; b) the presence of a lung abscess, empyema, or lung necrosis^6^; or c) a pleural drainage procedure was performed during the 30 days before hospitalization or during the hospital stay itself.^7^ |

Abbreviations: COPD, chronic obstructive pulmonary disease; ICD-10, International Classification of Diseases, Tenth Revision; VA, Veterans’ Affairs.

1. Hospitals with at least 12 months above the 25^th^ percentile for observed procalcitonin testing rates were selected for the analysis. We chose to limit our analysis to these hospitals because we speculated that the benefits of procalcitonin testing would most likely be realized in settings with experience interpreting the test
2. Eligible ICD-10 codes included codes for acute exacerbations of COPD (J44.0, J44.1) and for pneumonia (A48.1, B01.2, B05.2, B06.81, J09.X1, J10.0, J10.00, J10.01, J10.08, J11.0, J11.00, J11.08, J12, J12.0, J12.1, J12.2, J12.3, J12.8, J12.81, J12.89, J12.9, J13, J14, J15, J15.0, J15.1, J15.2, J15.20, J15.21, J15.211, J15.212, J15.29, J15.3, J15.4, J15.5, J15.6, J15.7, J15.8, J15.9, J16, J16.0, J16.8, J17, J18, J18.0, J18.1, J18.2, J18.8, J18.9). The mapping of these codes was borrowed from the following publication: King LM, Tsay SV, Hicks LA, Bizune D, Hersh AL, Fleming-Dutra K. Changes in outpatient antibiotic-prescribing for acute-respiratory illnesses, 2011-2018. *Antimicrob Steward Healthc Epidemiol* 2021; 1(1): e66.
3. Eligible antibacterial regimens were a macrolide (azithromycin or clarithromycin) plus an anti-pneumococcal beta-lactam (ampicillin-sulbactam, amoxicillin-clavulanate, cefdinir, ceftriaxone, cefotaxime, cefuroxime, or cefpodoxime).
4. Patient-admissions were excluded if any of the following antibacterial agents were prescribed: aminoglycosides, cephamycins, colistin, daptomycin, fosfomycin, lipoglycopeptides, nitrofurantoin, and tigecycline.
5. Patient-admissions were excluded if there were ICD-10 codes linked to the admission that indicated the presence of any of the following types of infections: biliary tract infections (cholangitis, cholecystitis, pancreatitis with infected necrosis); central nervous system infections (brain abscess and meningitis); endocarditis; intra-abdominal infections (appendicitis, colitis, diverticulitis, intestinal abscess, liver abscess, perforated bowel, peritonitis, rectal/anal abscess); osteo-articular infections (septic arthritis, osteomyelitis, orthopedic-device infection); skin and soft tissue infections; urinary tract infections (catheter-associated urinary tract infection, cystitis, pyelonephritis, prostatitis).
6. ICD-10 codes that indicated a complicated pneumonia include J85.0 (gangrene and necrosis of the lung); J85.1 and J85.2 (lung abscess); J85.3 (abscess of the mediastinum), and J86, J86.0, J86.9 (pyothorax).
7. Current Procedural Terminology codes were used to identify pleural drainage procedures, as classified by the Agency for Healthcare Research and Quality’s Clinical Classification Software: 32035-32036, 32550-32551, 32554-32557.

**Supplemental Table 3. Covariates included in the logistic regression model used to calculate propensity scores for the likelihood of receiving procalcitonin testing**

| **Covariate** | **Description** |
| --- | --- |
| Age | Age of the patient at the time of the acute-care admission. |
| Sex | Sex of the patient, based on administrative data. |
| Race | Race of the patient was categorized using administrate data as white, black, or other. |
| Infection type | Type of infection was categorized as an acute exacerbation of COPD, hospital-acquired pneumonia, or community-acquired pneumonia.. Community-acquired versus hospital-acquired was defined based on the timing of antibiotic initiation (≤ 48 hours from admission versus >48 hours after admission, respectively). |
| Comorbidities | Comorbidities were defined by using ICD-10 codes entered into the patient’s electronic medical record during the twelve months prior to admission or during the admission itself.^1,2^ The following comorbidities were included: congestive heart failure, valvular heart disease, neurologic disorders, chronic obstructive pulmonary disease, diabetes mellitus, acquired immunodeficiency syndrome, lymphoma, metastatic and non-metastatic cancer, alcohol disorder, dementia, and rheumatology diseases. |
| Immuno-suppression | A patient was categorized as immunosuppressed when at least one of the following criteria were present: a) receipt of chemotherapy during the 30 days prior to admission or during the hospital stay itself; b) receipt of an antirejection medication during the 3 months prior to admission or during the hospital stay itself; or c) having an inpatient or outpatient diagnosis code for an immunosuppressive condition within the 12 months before admission or during the hospitalization itself. Immunosuppressive conditions included diagnoses of leukemia, lymphoma, human immunodeficiency virus infection, bone marrow transplantation or solid organ transplantation. |
| Risk factors for antibiotic resistance | Three risk factors were each included as a separate covariate: a) receipt of dialysis during the prior 30 days or during hospitalization; b) admitted to the hospital from a nursing home; and c) receipt of wound care prior to admission. |
| MRSA nasal colonization | MRSA nasal screening was routinely performed upon admission to a VA acute-care bed during 2018 and 2019. During the COVID-19 pandemic, some VA medical centers chose to suspend MRSA screening. MRSA nasal test results were categorized as either a) positive or b) negative,/not done. |
| COVID-19 status | COVID-19 status was labeled as either a) positive or b) negative/not done. A patient was labeled as having COVID-19 if any COVID test was positive ≤ 14 days prior to admission or at any point during the hospital stay. |
| Urine Legionella antigen | Urine Legionella antigen tests completed within 24 hours before admission or during the hospital stay itself were captured. Patients were classified as being positive for Legionella if the urine antigen test was positive. |
| Modified APACHE score | The modified APACHE (Acute Physiology and Chronic Health Evaluation) score was used to measure patients’ severity of illness on hospital admission, as per a previously published protocol.^3^ Missing values for the APACHE score were assumed to be normal; missing values were uncommon (<5%) except for albumin and bilirubin, which were missing for roughly 25% of admissions. A modified APACHE score was used because certain data elements (Glasgow Coma Scale, urinary output, acid-base status, mechanical ventilation, and PaO_2_) were not available for patients not admitted to the intensive care unit. |
| Hospital unit | Hospital unit was defined at the point of hospitalization when cases and controls were matched: intensive care unit (ICU) or non-ICU. |
| Vital signs | Vital signs (temperature, heart rate, blood pressure, and respirations) were measured at the point of hospitalization when cases and controls were matched. |
| White blood cell count | White blood cell count was measured at the point of hospitalization when cases and controls were matched. |
| Serum creatinine | Serum creatinine was measured at the point of hospitalization when cases and controls were matched. |
| Antibiotic class | Antibiotic classes were measured at the point of hospitalization when cases and controls were matched. Classes included cephalosporins, beta-lactam/beta-lactamase inhibitors, carbapenems, fluoroquinolones, glycopeptides, macrolides, tetracyclines, and other. |

Abbreviations: APACHE, Acute Physiology and Chronic Health Evaluation; COPD, chronic obstructive pulmonary disease; COVID-19, coronavirus disease of 2019; ICD-10, International Classification of Diseases, Tenth Revision; ICU, intensive care unit; MRSA, methicillin-resistant *Staphylococcus aureus*.

1. Elixhauser  A, Steiner  C, Harris  DR, Coffey  RM.  Comorbidity measures for use with administrative data. *Med Care*. 1998;36(1):8-27.
2. Quan  H, Sundararajan  V, Halfon  P,  et al.  Coding algorithms for defining comorbidities in *ICD-9-CM* and *ICD-10* administrative data. *Med Care*. 2005;43(11):1130-1139.
3. Knaus  WA, Wagner  DP, Draper  EA,  et al.  The APACHE III prognostic system: risk prediction of hospital mortality for critically ill hospitalized adults. *Chest*. 1991;100(6):1619-1636.

| **Supplemental Table 4. Characteristics of hospitals with and without on-site procalcitonin testing**^1^ | | |  |
| --- | --- | --- | --- |
|  | **Procalcitonin available**  **(n= 81)** | **Procalcitonin not available**  **(n =44)** | **p-value** |
| Hospital complexity, n (%) |  |  |  |
| 1a, 1b, or 1c | 63 (77.8) | 29 (65.9) | p=0.15 |
| Urban, n (%) | 76 (93.8) | 34 (77.3) | p<0.01 |
| On-site ID consultation service, n (%) | 69 (85.2) | 32 (72.7) | p=0.09 |
| Stewardship resources and strategies, n (%) |  |  |  |
| ASP provider time commitment^2^ |  |  |  |
| 26-100% | 22 (27.2) | 5 (11.6) | P<0.05 |
| ASP pharmacist time commitment^3^ |  |  |  |
| 51-100% | 49 (60.5) | 16 (37.2) | p=0.01 |
| Frequency at which ASP champions meet |  |  |  |
| Daily | 28 (36.8) | 13 (31.0) | p=0.37 |
| Several times per week | 21 (27.6) | 10 (23.8) |  |
| Clinical pathway for CAP and HAP | 54 (66.7) | 23 (52.3) | p=0.11 |
| Prospective audit and feedback on antibiotic day 1-2 |  |  |  |
| At least 3-4 times/week | 69 (85.2) | 40 (90.9) | p=0.36 |
| Prospective audit and feedback on antibiotic day 4 |  |  |  |
| At least 3-4 times/week | 63 (77.8) | 35 (79.6) | p=0.82 |
| Policy or process to assess antibiotic duration at hospital discharge | 73 (90.1) | 31 (70.5) | p<0.01 |
| Restricted use of ciprofloxacin, levofloxacin, and/or moxifloxacin | 63 (77.8) | 33 (75.0) | p=0.73 |
| Procalcitonin utilization, mean (SD) |  |  |  |
| Number of months test was available | 38.5 (11.3) | 2.4 (3.4) |  |
| Number of tests/month | 60.9 (59.4) | 2.5 (3.9) |  |
| Data are presented as mean (standard deviation) or number (%). Percentages may not sum to 100% due to rounding. Abbreviations: ID, infectious disease; ASP, antibiotic stewardship program; CAP, community-acquired pneumonia; HAP, hospital-acquired pneumonia.   1. Data on the antibiotic stewardship resources and processes were obtained from a mandatory survey of all VA hospitals conducted between 10/20/2020 and 11/10/2020. 2. Data not reported from six sites 3. Data not reported from one site 4. Data not reported from seven sites. | | |  |
